# Four novel mutations identified in the *COL4A3*, *COL4A4* and *COL4A5* genes in 10 families with Alport syndrome

**DOI:** 10.1186/s12920-024-01953-0

**Published:** 2024-07-08

**Authors:** Duocai Wang, Meize Pan, Hang Li, Minchun Li, Ping Li, Fu Xiong, Hongbo Xiao

**Affiliations:** 1 Department of Medical Genetics, School of Basic Medical Sciences, Southern Medical University, Guangzhou, Guangdong China; 2 Department of Nephrology, Peking University Shenzhen Hospital, Futian, Shenzhen, Guangdong 518036 China; 3 Department of Urology, Peking University Shenzhen Hospital, Futian, Shenzhen, Guangdong China; 4 Department of Pathology, Peking University Shenzhen Hospital, Futian, Shenzhen, Guangdong China; 5 Guangdong Provincial Key Laboratory of Single Cell Technology and Application, Guangzhou, Guangdong China; 6 Department of Fetal Medicine and Prenatal Diagnosis, Zhujiang Hospital, Southern Medical University, Guangzhou, China

**Keywords:** *COL4A3*, *COL4A4*, *COL4A5*, Alport syndrome

## Abstract

**Background:**

Alport syndrome (AS) is an inherited nephropathy caused by mutations in the type IV collagen genes. It is clinically characterized by damage to the eyes, ears and kidneys. Diagnosis of AS is hampered by its atypical clinical picture, particularly when the typical features, include persistent hematuria and microscopic changes in the glomerular basement membrane (GBM), are the only clinical manifestations in the patient.

**Methods:**

We screened 10 families with suspected AS using whole exome sequencing (WES) and analyzed the harmfulness, conservation, and protein structure changes of mutated genes. In further, we performed in vitro functional analysis of two missense mutations in the *COL4A5* gene (c.2359G > C, p.G787R and c.2605G > A, p.G869R).

**Results:**

We identified 11 pathogenic variants in the type IV collagen genes (*COL4A3*, *COL4A4* and *COL4A5*). These pathogenic variants include eight missense mutations, two nonsense mutations and one frameshift mutation. Notably, Family 2 had digenic mutations in the *COL4A3* (p.G1170A) and *UMOD* genes (p.M229K). Family 3 had a digenic missense mutation (p.G997E) in *COL4A3* and a frameshift mutation (p.P502L fs*151) in *COL4A4*. To our knowledge, four of the 11 mutations are novel mutations. In addition, we found that *COL4A5* mutation relation mRNA levels were significantly decreased in HEK 293 T cell compared to control, while the cellular localization remained the same.

**Conclusions:**

Our research expands the spectrum of *COL4A3*-5 pathogenic variants, which is helpful for clinical and scientific research.

**Supplementary Information:**

The online version contains supplementary material available at 10.1186/s12920-024-01953-0.

## Introduction

Alport syndrome (AS) is the first inherited kidney disease for which the genetic basis has been established [1], The clinical features of AS are eye, ear and kidney damage, which are caused by mutations in the type IV collagen genes [2]. Pathogenic variants in the *COL4A3*, *COL4A4* and *COL4A5* genes result in defects in the synthesis of the α3, α4 and α5 chains of type IV collagen. This prevents the correct assembly of the glomerular basement membrane (GBM) collagen network and leads to AS [3]. The modes of inheritance of AS are X-linked AS (XLAS), autosomal recessive AS (ARAS) and autosomal dominant AS (ADAS) [4]. The XLAS version is caused by variants in the *COL4A5* gene and accounts for approximately 85% of cases. The ARAS and ADAS versions are caused by variants in the *COL4A3* or *COL4A4* genes and account for approximately 15% of cases [5].

Electron microscopy (EM) of renal biopsies from AS patients show characteristic abnormalities in the GBM. These manifest as irregular thinning and varying degrees of GBM thickening, were the dense layer splits and fragments into several strands, to form a 'basket weave' pattern [6]. Classic AS is characterized by hematuria with progressive proteinuric nephropathy, GBM abnormalities, hearing loss and ocular abnormalities [7]. It accounts for at least 1–2% of all patients who receive kidney replacement therapy (KRT) [8]. The clinical course in female carriers is variable and is usually milder than in males, due to inactivation of the X chromosome [9]. Women with XLAS can develop proteinuria but only 30% of female patients will develop end-stage renal disease by the age of 60 [10, 11]. In contrast, the disease is characterized by end-stage renal failure in 95% of male patients. It has also been reported that the renal phenotype in males with ADAS is significantly less severe than in males with ARAS or XLAS [12].

Although we have some understanding of the mechanisms and clinical phenotypes of AS, it is difficult to distinguish thin basement membrane nephropathy (TBMN) from the initial stages of AS, particularly when persistent hematuria and thin GBM lesions are the only manifestations. However, molecular analysis of the type IV collagen genes can provide information for diagnosis, prenatal diagnosis and genetic counseling [13].

In this study, four novel pathogenic variants in the *COL4A3*, *COL4A4* and *COL4A5* genes were identified in 24 patients from 10 families with AS, using whole exome sequencing (WES), histological testing and bioinformatics prediction. The missense mutation in *COL4A5* (p.G869R, p.G787R) was validated by in vitro functional analysis.

## Methods

### Patients

This study was conducted in accordance with the Declaration of Helsinki (revised 2013) and was approved by the Peking University Shenzhen Hospital Institutional Review Board. Written informed consent was obtained from all participants or their guardians. Clinical data were missing for some of the patients.

We investigated 24 patients (17 female and seven male, age range: 1 to 70 years) from 10 unrelated families with suspected AS or TBMN, recruited in Peking University Shenzhen Hospital. The diagnostic criteria for AS met the following conditions: organ damage and kidney GBM EM abnormalities, mutations in a type IV collagen gene or abnormal expression of type IV collagen, organ damage with the presence of persistent hematuria and/or proteinuria, treble sensory deafness and positive family history of diagnostic ophthalmic signs [14, 15]. Kidney damage was assessed with tests for hematuria, proteinuria and glomerular filtration rate, where the estimated glomerular filtration rate (eGFR) was calculated by the CKD Epidemiology Collaboration creatinine equation.

Biopsies were taken from kidney tissue from nine patients with hematuria plus proteinuria. They were examined by light microscopy (Hematoxylin–eosin staining (HE), Periodic Acid-Schiff stain (PAS), Masson’s Trichrome Staining, periodic acid-silver metheramine (PASM) and EM and showed extensive, irregular thickening of the GBM, with longitudinal tearing of the dense layer to form a lamellar or basket-like pattern. This was observed with or without diffuse thinning and uneven thickness of the GBM, or infiltration of foam cells in the renal interstitium.

### Mutation screening

Genomic DNA was extracted from the peripheral blood from the patient and family members using a standard phenol/chloroform extraction method. Samples were analyzed by WES and Sanger sequencing to screen for mutations in all exons of the *COL4A3*, *COL4A4* and *COL4A5* genes.

### Bioinformatics

All variants were assessed for pathogenicity using the by Mutation Taster, Mutation Assessor, Fathmm, Predictsnp, polymorphism phenotyping (PolyPhen), population databases dbSNP and disease databases Clinvar. We used I-TASSER to predict and construct the protein structure models of the wild-types and mutants. The structural changes between wild-type and mutant were then compared. The amino acids affected by the mutations were analyzed for species conservation using PolyPhen-2.

### Functional analysis of mutant *COL4A5*

#### Plasmid constructs and mutagenesis

The coding sequence of the *COL4A5* gene was cloned into the HindIII and SacII sites of the pEGFP N1 vector to construct the wild-type *COL4A5*-WT-EGFP overexpression vector. This was used as a template to construct the mutant eukaryotic expression vectors, *COL4A5*-G787R-EGFP and *COL4A5-*G869R-EGFP. The following primer pairs were used for PCR analysis: c.2359G > C forward, 5'‑CCTCCGGGTCCTCCACGACGCACTGGCTTAG-3’; c.2359G > C reverse, 5'‑CTAAGCCAGTGCGTCGTGGAGGACCCGGAGG‑3’. c.2605G > A forward, 5'‑AGTCCAGGGATCCCCAGAGCACCTGGTCCTA‑3’; c.2605G > A reverse, 5'‑TAGGACCAGGTGCTCTGGGGATCCCTGGACT‑3'. The following thermocycling conditions were used for PCR: Initial denaturation for 2 min at 94 °C; followed by 17 cycles of denaturation (10 s; 98 °C) and extension (9 min 48 s; 68 °C); and a final extension for 10 min at 68 °C.

#### Cellular localization

Human embryonic kidney (HEK) 293 T cells, at 70% confluence (Guangzhou Saiku Biotechnology Co., Ltd.), were grown in DMEM (Invitrogen; Thermo Fisher Scientific, Inc.) and supplemented with 10% FBS (Gibco; Thermo Fisher Scientific, Inc.), at 37 °C. They were then transiently transfected using Lipofectamine™ 2000 (Invitrogen; Thermo Fisher Scientific Inc.), with 2.5 μg of the empty vector (pEGFP N1) or the recombinant plasmids that contained wild-type (*COL4A5*-WT-EGFP) or mutant *COL4A5* genes (*COL4A5*-G787R-EGFP or *COL4A5*-G869R-EGFP). After 24 h of transfection, cells were washed three times with PBS (pH7.4) and fixed for 30 min with 4% paraformaldehyde (Sigma-Aldrich). Following fixation, the paraformaldehyde was discarded and samples were washed three times with PBS. Cells were then incubated in 0.1% Triton X-100 (Thermo Fisher Scientific, Massachusetts, USA), to increase the permeability of the cytomembrane, and were subsequently washed with PBS. To observe the subcellular location of the *COL4A5* protein, nuclei were stained with 4’, 6-diamidino-2-phenylindole (DAPI; Sigma) for 10 min and then washed three times with PBS. Fluorescence microscopy (LSM 880; Carl Zeiss AG) was used to visualize the fluorescence signal from the transfected cells, at 2000X magnification.

### RNA analysis

To determine the expression of wild-type and mutant *COL4A5*, recombinant constructs were transfected into 293 T cells using Lipofectamine™ 2000 (Invitrogen; Thermo Fisher Scientific, Inc.). After 24 h of transfection, total RNA was isolated using Trizol reagent (Invitrogen) and reverse transcribed into cDNA using the PrimeScript™ RT reagent kit (Takara, Dalian, China). Real-time PCR was used to measure the relative mRNA levels of wild-type and mutant *COL4A5* genes using the 2 × RealStar Green Fast Mixture (GenStar, Beijing, China). We used GAPDH as the reference gene to normalize *COL4A5* expression. The *COL4A5* primers were as follows: forward: 5’-CAAAAGGTGATCGTGGTTTCCC-3’; reverse:5’-GTCCAGGTTGTCCATTTGGTC-3’. The GAPDH primers were as follows: forward: 5’-GTGAAGGTCGGAGTCAACG-3’; reverse: 5’-TGAGGTCAATGAAGGGGTC-3’. Gene expression levels were calculated using the 2^−ΔΔCT^ method. To confirm the reproducibility of the results, transfection and real-time PCR assays were repeated three times.

### Statistical analysis

Statistical analysis was performed using GraphPad Prism software (GraphPad Software Inc., San Diego, CA, USA). Statistical significance between two groups was determined using the independent samples t test. Data are presented as the mean ± SEM and the experiments were repeated three times. A *p* value < 0.05 was considered to be statistically significant. *p < 0.05, **p < 0.01, ***p < 0.001 and ****p < 0.0001.

## Results

### Clinical phenotype

The 24 AS patients included in this study had been treated at Peking University Shenzhen Hospital for progressive hematuria. The patients had progressive hematuria and urinary protein, to varying degrees, and a decreased eGFR. Interestingly, the clinical phenotypes of patients varied greatly, even between patients with the same mutation (Table 1). It should be emphasized that, in this study, TBMN and AS were not classed as different diseases in the diagnostic findings. We adopted the AS classification of Clifford E. Kashtan et al. [16], which considers TBMN as a description of the lesion, rather than a diagnostic finding, and classifies hematuria and TBMN as AS. The raw clinical data are given in Table 1. For the purpose of this study, TBMN diagnosed by physicians, in a clinical setting, was classed as AS.

**Table 1:** Mutations in the type IV collagen genes (COL4A3, COL4A4 and COL4A5), identified in 22 patients from 10 families

**Family**	**Member**	**Gender**	**Age of diagnosis**	**Disease**	**The mode of inheritance**	**EM GBM changes**		**Hematuria**	**Proteinuria**	**eGFR(ml/min)**	**Mutations**				
						**Diffuse thinning**	**Splitting/Uneven thickness/ Stratification**				**Gene**	**Zygosity**	**Exon**	**Nucleotide change**	**Amino acid change**
1	II-1	F	40	AS	AD	YES	YES	58/ul	+	62	COL4A3	Het	21	c.1213G > T	p.Glu405*
2	II-1	M	52	AS	AD			2–5/HP	normal	68	COL4A3	Hom	40	**c.3509G > C**	**p.Gly1170Ala**
	III-1	F	25	AS + ADTKD		YES	YES	1500/ul	+	65.2	COL4A3	Het	40	**c.3509G > C**	**p.Gly1170Ala**
											UMOD	Het	3	**c.686 T > A**	**p.Met229Lys**
3	I-1	M	64	normal	AR			normal	normal	normal	COL4A4	Het	22	c.1505del	p.pro502Leufs*151
	I-2	F	61	AS				normal	+ +	27.2	COL4A3	Het	36	c.2990G > A	p.Gly223Val
	II-1	F	40	AS						33	COL4A3	Het	36	c.2990G > A	p.Gly977Glu
	II-2	F	38	AS				1500/ul	-	130	COL4A4	Het	22	c.1505del	p.pro502Leufs*151
	II-3	F	35	AS		YES	YES	+ +	+	47	COL4A3	Het	36	c.2990G > A	p.Gly977Glu
											COL4A4	Het	22	c.1505del	p.pro502Leufs*151
	II-4	M	32	AS				457/ul	+ +	92.2	COL4A3	Het	36	c.2990G > A	p.Gly977Glu
											COL4A4	Het	22	c.1505del	p.pro502Leufs*151
	III-1	M	7	normal				normal	normal	normal	COL4A4	Het	22	c.1505del	p.pro502Leufs*151
4	II-5	F	70	AS	AD			+	+	41	COL4A4	Het	11	**c.668G > T**	**p.Gly223Val**
	III-2	F	49	AS		YES	NO	147/ul	+	86	COL4A4	Het	11	**c.668G > T**	**p.Gly223Val**
5	II-1	F	25	AS	AR(Digenic)	YES	YES	+ +	+	40.9	COL4A4	Hom	14	**c.826G > A**	**p.Gly276Arg**
6	I-1	M	57	AS	AD			+	-	77	COL4A4	Het	21	c.1396G > A	p.Gly466Arg
	II-1	F	29	AS		YES	YES	591/ul	+ +	108	COL4A4	Het	21	c.1396G > A	p.Gly466Arg
7	Proband	F	29	AS	AD	YES	YES	103/ul	+ + +	107	COL4A4	Het	44	c.4129C > T	p.Arg1377*
8	I-1	M		-	AD			86/ul	+		COL4A4	Het	48	c.5044C > T	p.Arg1682Trp
	II-1	F	28	AS		YES	YES	3–6/HP	+ +		COL4A4	Het	48	c.5044C > T	p.Arg1682Trp
9	II-1	M	34	AS	XL	YES	YES	146/ul	+	59	COL4A5	Hem	29	c.2359G > C	p.Gly787Arg
	II-2	F	69					+	+ +		COL4A5	Het	29	c.2359G > C	p.Gly787Arg
	III-1	F	11	AS				1158/ul	+ +	90	COL4A5	Het	29	c.2359G > C	p.Gly787Arg
10	I-2	F	58	AS	XL			+	+ +		COL4A5	Het	31	c.2605G > A	p. Gly869Arg
	II-2	F	32	AS				+	+ +		COL4A5	Het	31	c.2605G > A	p. Gly869Arg
	III-1	F	1	AS				+	+	110	COL4A5	Het	31	c.2605G > A	p. Gly869Arg

The normal reference values for hematuria and proteinuria are negative, while the normal reference value for eGFR is greater than 80 ml/min. The reference value of hematuria was > 3/HP or > 13/ul

Previously undescribed mutations are shown in bold

*F* Female, *M* Male, *EM GBM changes* Changes in the glomerular basement membrane, as seen under an electron microscope, *AS* Alport syndrome, *ADTKD* Autosomal dominant tubulointerstitial kidney disease

### Pathological manifestations

**Fig. 1:**
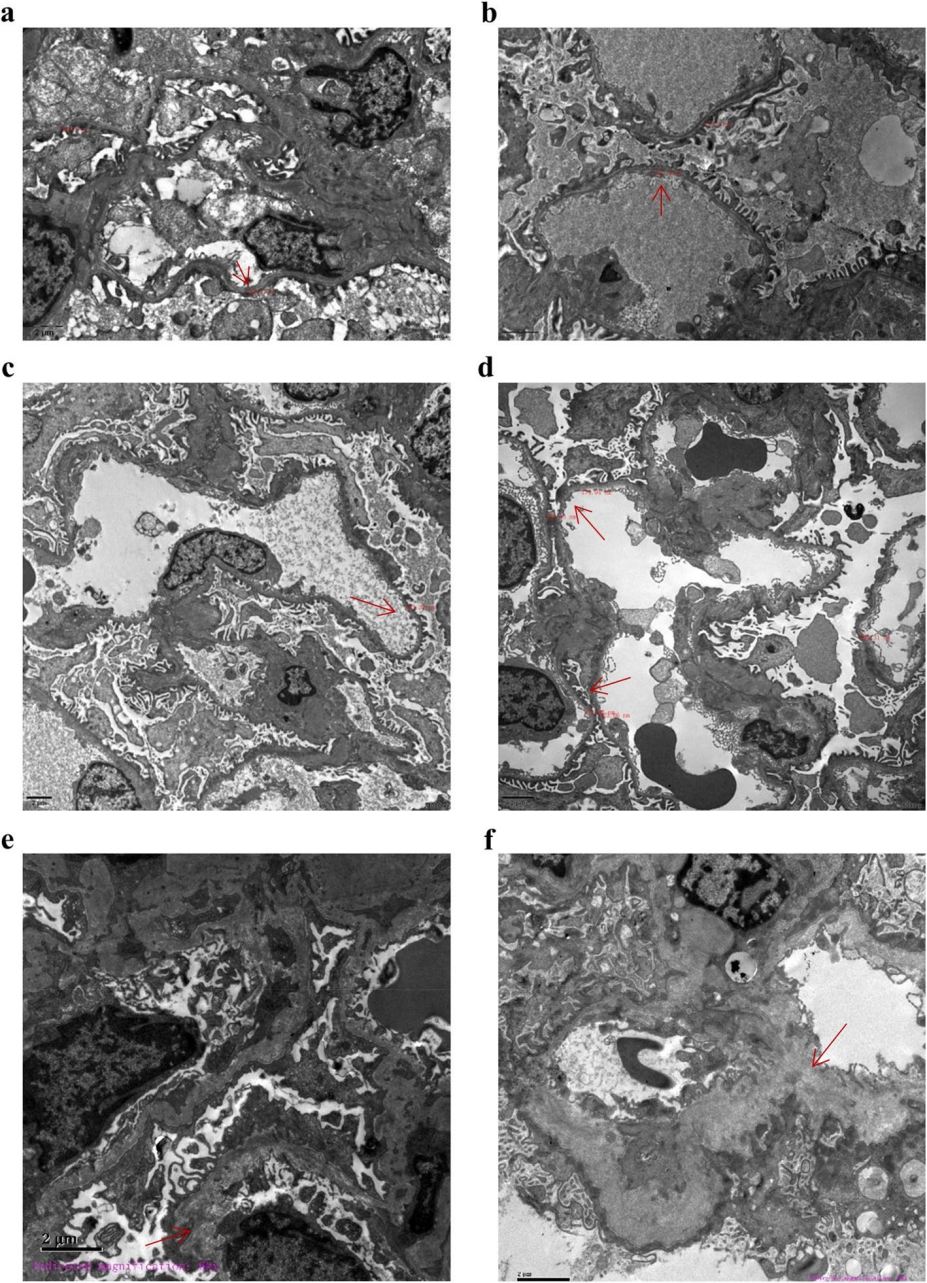
Reprocessed electron micrographs of a renal biopsy from a patient. The red arrow indicates that the GBM is thinner or of different thickness. **a** Patient II-1 of Family 1. **b** Patient III-1 of Family 2. **c** Patient III-2 of Family 4. **d** Patient II-1 of Family 5. **e** Proband of Family 6. **f** Patient II-1 of Family 8. Scale bar represents 2 μm

The renal biopsy results from nine patients with hematuria and proteinuria showed that the basement membrane was thinner, with or without stratification, and the podocytes has disappeared in some patients. The EM of the renal biopsies from Family 1, II -1, and Family 7, III-1 showed that the glomerular capillary endothelial cells of these patients had obvious vacuolar degeneration. The thickness of the basement membrane was approximately 140–560 nm. The segmental basement membrane was loose and stratified (Fig. 1a, e). Visceral epithelial cells were swollen with vacuolar degeneration. The podocytes was fused segmental. Mesangial cells and stroma proliferated, and no electron dense deposits were observed.Renal tubulointerstitial: vacuolar degeneration of renal tubular epithelial cells. There were no specific renal interstitial lesions (Fig. 1a). The EM of the renal biopsy from proband III-1 of Family 2 showed that the GBM was diffusely thinner and its thickness was less than 200 nm (Fig. 1b). It is worth emphasizing that both PAS and PASM staining results of III-1 of Family 2 show that crystalline material deposition can be seen in the renal tissue, and the deposition of crystalline substances in renal tissue is likely due to uric acid (Supplementary1). Visceral epithelial cells were swollen with vacuolar degeneration. The podocytes was fused segmental, and proliferation of mesangial cells and stroma. The patients in Family 4 and Family 6 were diagnosed as having TBMN, as their renal biopsy EM results showed that the GBM was diffusely thinner, with a thickness of less than 200 nm. There was no obvious dense layer stratification (Fig. 1c-d). The results of the renal biopsy for Family 9, II-1, also showed that the dense layer of the GBM was thickened and some areas were lacerated and arachnoid (Fig. 1f).

### Bioinformatics analysis of mutation harmfulness

**Fig. 2:**
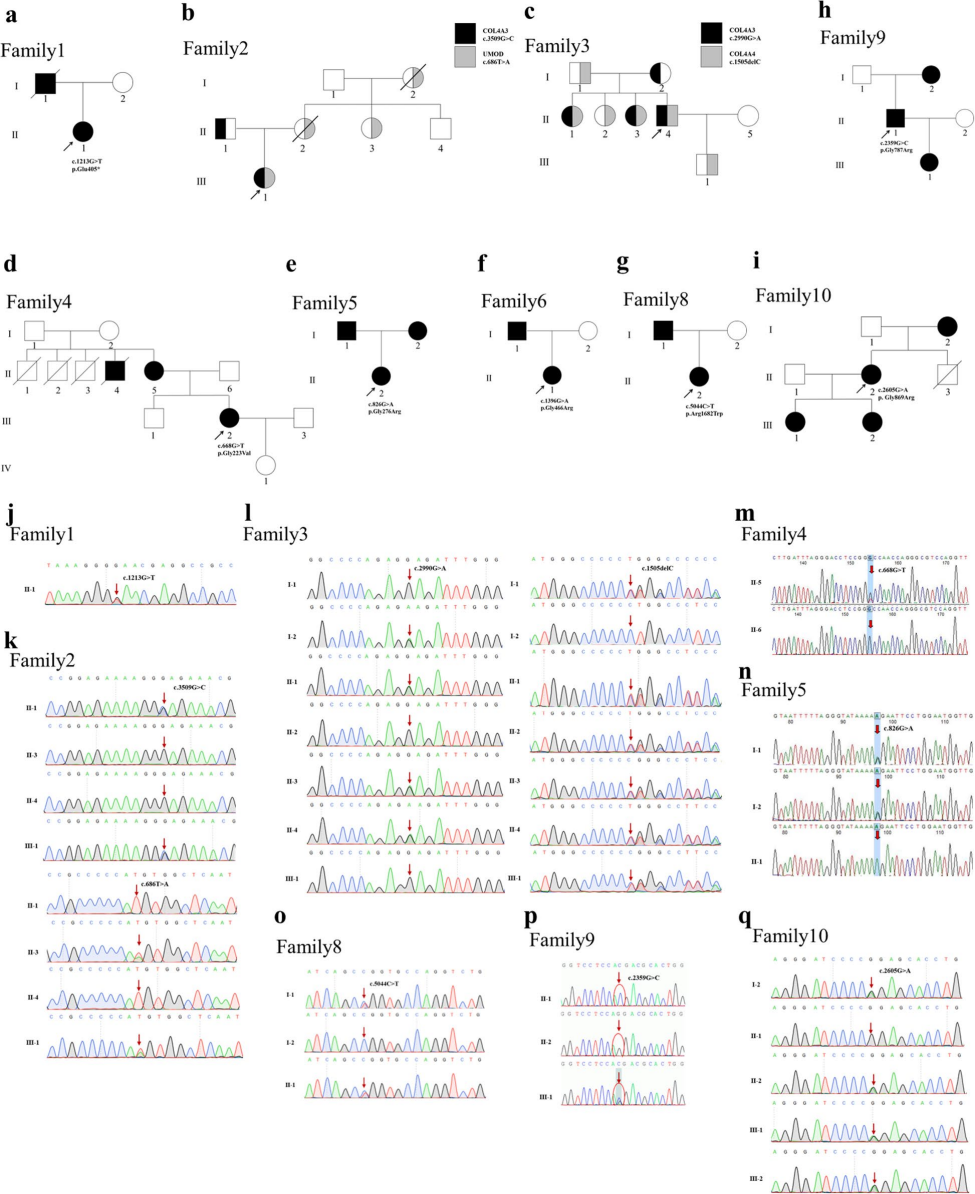
Family pedigree and Sanger sequencing results of affected families with type IV collagen gene mutations. Males are marked as squares and females as circles. An arrow indicates the proband and the black symbols indicate the affected individuals. Het, Heterozygote. **a** Family 1, *COL4A3*, c.1213G > T. **b** Family 2, *COL4A3*, c.3509G > C. **c** Family 3, *COL4A3*, c.2990G > A, *COL4A4*, c.1505delC. **d** Family 4, *COL4A4*, c.668G > T. **e** Family 5, *COL4A4*, c.826G > A. **f** Family 6, *COL4A4*, c.1396G > A. **g** Family 7, *COL4A4*, c.4129C > T. **h** Family 8, *COL4A4*, c.5044C > T. **i** Family 9, *COL4A5*, c.2359G > C. **j** Family 10, *COL4A5*, c .2605G > A. **k**-**r** The sequence chromatograms below the family pedigrees show the Sanger sequencing results for the family members

**Fig. 3:**
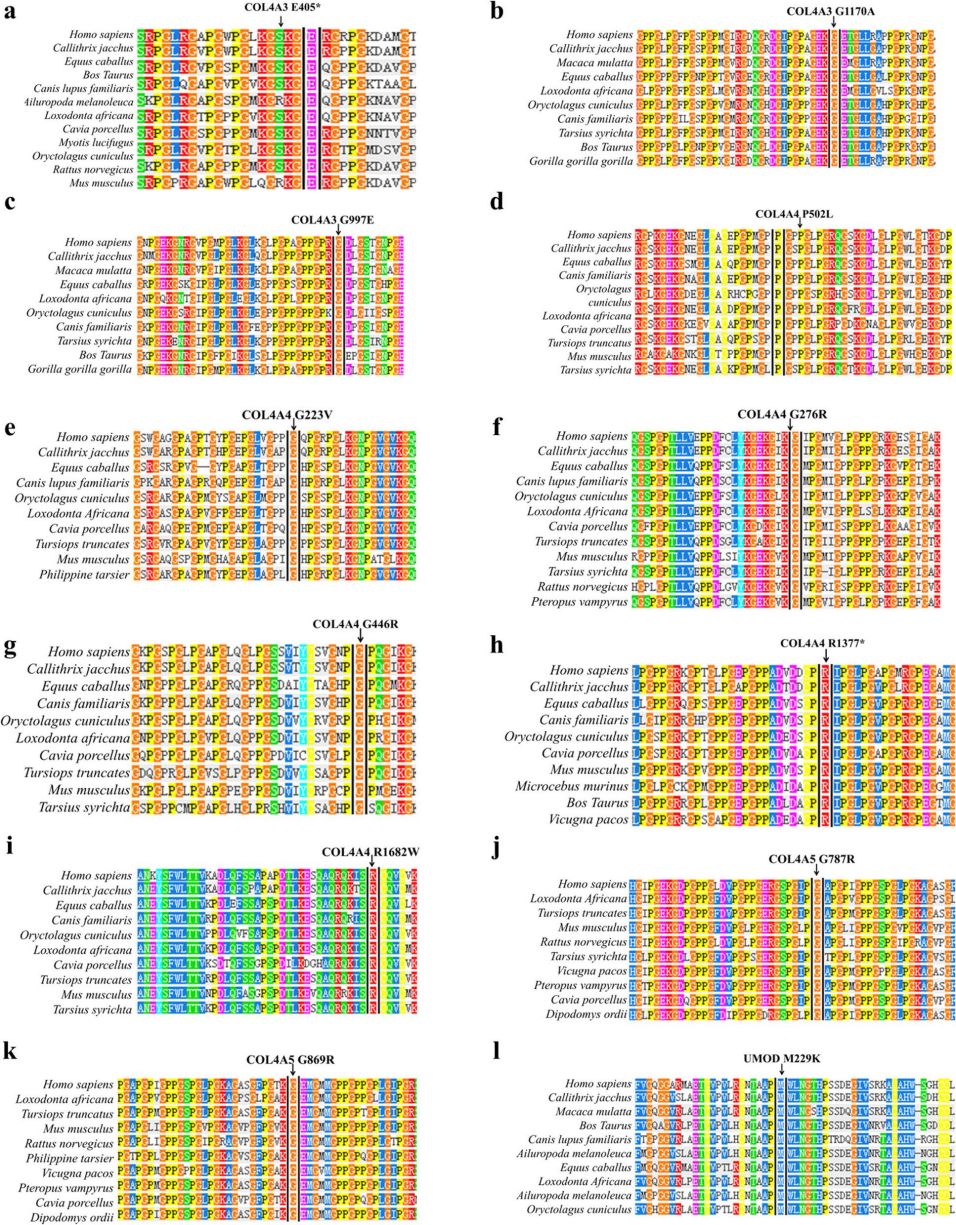
Conservation analysis of abnormal variation, using Polyphen-2. The mutated amino acids were highly conserved among species. **a**-**c**, *COL4A3* mutations. **d**-**i**, *COL4A4* mutations. **j**, **k**, *COL4A5* mutations. **l**, *UMOD* mutations

**Fig. 4:**
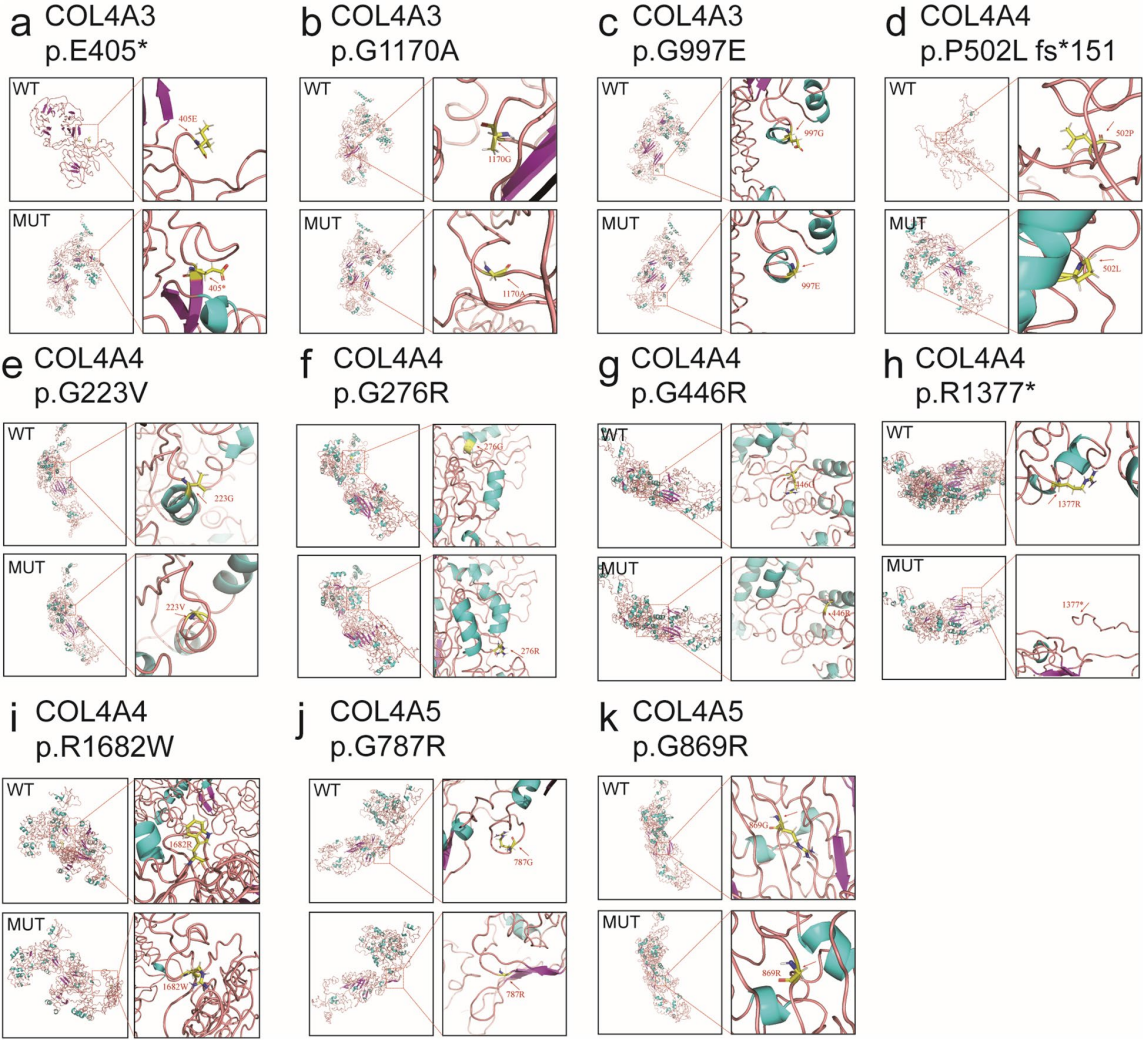
Prediction of wild-type and mutant protein structures by I-TASSER. **a**-**c**, *COL4A3* mutations. **d**-**i**, *COL4A4* mutations. **j**, **k**, *COL4A5* mutations. The red dotted line is the locally amplified protein structure

Blood samples were collected for genetic screening and the pedigrees were drawn using the detailed family information (Fig. 2). We used the sequencing results and pedigrees to analyze the inheritance of the mutations. For example, the *COL4A3* missense mutation (c.2990G > A, p.G997E) in Family 3, II-4, was inherited from the mother, I-2, whilst the *COL4A4* frameshift mutation (c.1505del, p.P502Lfs * 151) was inherited from the father, I-1. (Fig. 2c, l). The mutated amino acids were highly conserved among species (Fig. 3). Using the I-TASSER standard, we analyzed the two-dimensional and three-dimensional changes in structure between the wild-type and mutant proteins. Changes were found in the three-dimensional structure of each protein. The more obvious changes, based on I-TASSER model, were caused by the *COL4A3* nonsense mutation (p.E405 *), the *COL4A4* frameshift mutation (p.P502Lfs * 151) and the *COL4A4* nonsense mutation (p.R1377 *) (Fig. 4a, d, h). For other mutations, obvious local changes can be seen in the enlarged image on the right (Fig. 4b, c, e, f, g, i, j, k). The prediction results for mutation harmfulness, using multiple databases, showed that the 12 mutations found in this study, which included the *UMOD* missense mutation (c.686 T > A, p.Met229Lys), were predicted to be pathogenic, likely pathogenic or uncertain significance (Table 2).

**Table 2:** Prediction of harmfulness of gene mutations

Family	Gene	Location(GRCh37.p13)	Mutation		Polyphen2	Mutation Taster	Mutation Asscssor	Fathmm	Predictsnp	Clinvar	dbSNP ID	MAF	ACMG criteria
			**DNA**	**Amino Acid**									
1	*COL4A3*	*chr2:228,128,558*	c.1213G > T	p.Glu405*	-	Disease causing	Stop gain	Damaging	Stop gain	Stop gain	rs2070736908	0.000031	Pathogenic
2	*COL4A3*	*chr2:228,159,770*	**c.3509G > C**	**p.Gly1170Ala**	Probably damaging	Disease causing	High	Damaging	Deleterious	-	-	-	Likely pathogenic
	*UMOD*	*chr16:20,359,937*	**c.686 T > A**	**p.Met229Lys**	Probably damaging	Disease causing	-	-	-	-	-	-	Likely pathogenic
3	*COL4A3*	*chr2:228,154,724*	c.2990G > A	p.Gly977Glu	Probably damaging	Disease causing	High	Damaging	Deleterious	Uncertain/conflicting	rs1553762113	-	Pathogenic
	*COL4A4*	*chr2:227,953,487*	c.1505delC	p.pro502Leufs*151	-	Disease causing	-	-	-	-	-	-	Pathogenic
4	*COL4A4*	*chr2:227,973,574*	**c.668G > T**	**p.Gly223Val**	Probably damaging	Disease causing	-	Damaging	-	-	-	-	Likely pathogenic
5	*COL4A4*	*chr2:227,967,904*	**c.826G > A**	**p.Gly276Arg**	Probably damaging	Disease causing	-	Damaging	Deleterious	-	-	-	Uncertain significance
6	*COL4A4*	*chr2:227,954,647*	c.1396G > A	p.Gly466Arg	Probably damaging	Disease causing	-	Damaging	-	Pathogenic	-	0.00019968	Pathogenic
7	*COL4A4*	*chr2:227,886,850*	c.4129C > T	p.Arg1377*	-	Disease causing	-	-	-	Pathogenic	rs559719653	0.000028	Pathogenic
8	*COL4A4*	*chr2:227,872,070*	c.5044C > T	p.Arg1682Trp	Probably damaging	Disease causing	-	Damaging	-	Uncertain/conflicting	-	0.000012	Pathogenic
9	*COL4A5*	*chrX:107,850,086*	c.2359G > C	p.Gly787Arg	Probably damaging	Disease causing	High	Damaging	Deleterious	-	rs1603293605	-	Pathogenic
10	*COL4A5*	*chrX:107,863,584*	c.2605G > A	p. Gly869Arg	Probably damaging	Disease causing	High	Damaging	Deleterious	Pathogenic/Likely pathogenic	-	-	Likely pathogenic

^a^Polyphen-2, prediction of functional effects of human nsSNPs, http://genetics.bwh.harvard.edu/

^b^Mutation Taster, https://www.mutationtaster.org/

^c^Mutation Assessor, http://mutationassessor.org/

^d^Fathmm, http://fathmm.biocompute.org.uk/

^e^Predict SNP, https://loschmidt.chemi.muni.cz/predictsnp2/

fClinVar, https://www.ncbi.nlm.nih.gov/clinvar

^g^dbSNP, https://www.ncbi.nlm.nih.gov/snp

^h^MAF was derived from dbSNP and gnomAD databases

Previously undescribed mutations are shown in bold

### Functional analysis

**Fig. 5:**
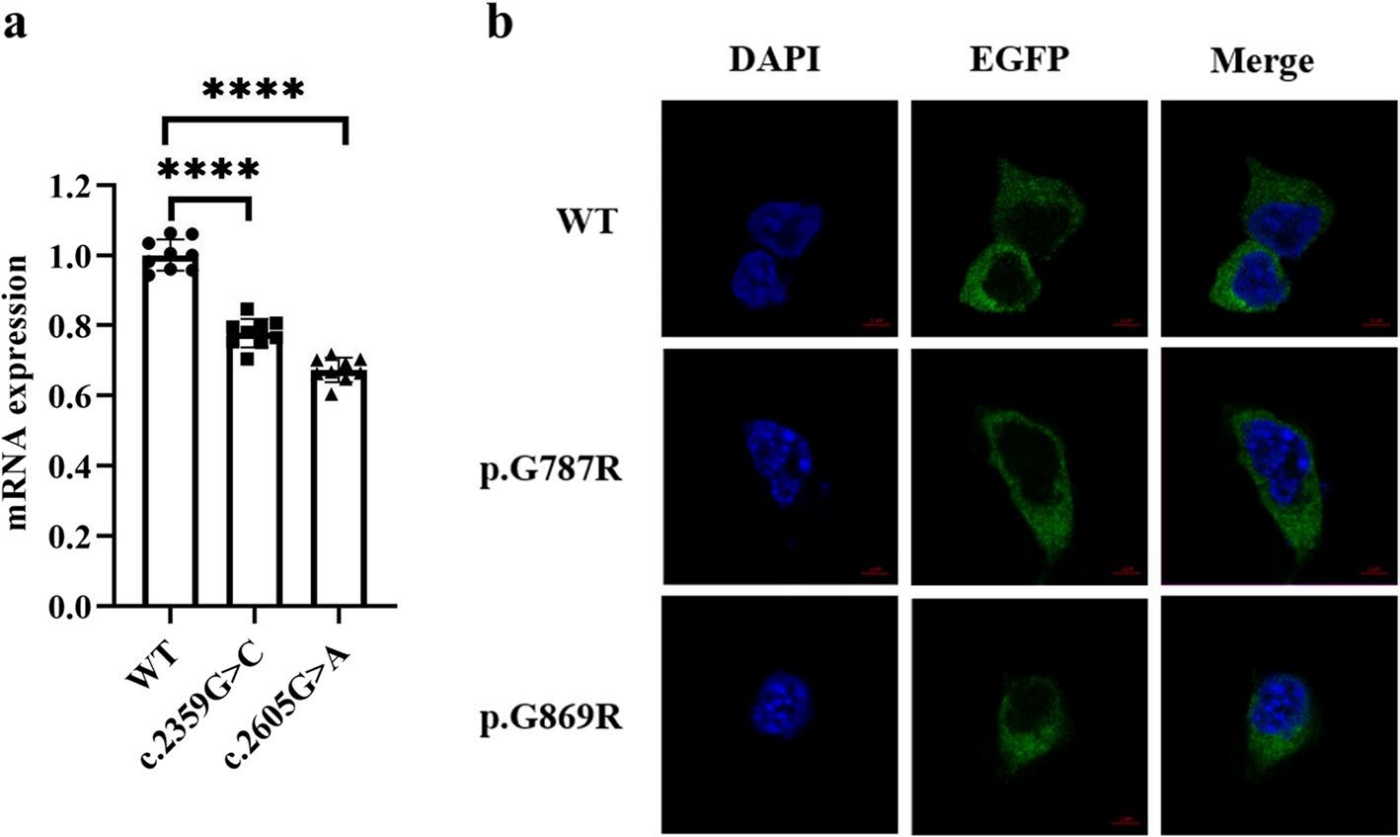
Effect of a mutation on *COL4A5* function. **a** mRNA level of *COL4A5* in HEK293T cells. ****p < 0.0001. **b** Subcellular localization of *COL4A5* in HEK293 cells. Confocal images of EGFP (green), DAPI nuclear staining (blue) and merged signals. Scale bar represents 5 μm

We analyzed the effects of *COL4A5* mutation on its mRNA expression and subcellular localization in HEK293T cell. The results showed that there were significant differences in mRNA levels. Specifically, when compared with the wild-type, the mRNA levels decreased significantly in cells with both the c.2359G > A mutation and the c.2605G > A mutation (Fig. 5a). Nevertheless, there was no significant difference in subcellular localization between the wild-type and mutant group and *COL4A5* was expressed in the cytoplasm (Fig. 5b).

## Discussion

In this study, clinical, pathological and genetic information was provided for 24 patients from 10 families. Ten pathogenic variants were identified in type IV collagen genes (*COL4A3*, *COL4A4* and *COL4A5*). Four of the mutations were novel, including a novel *COL4A3* missense mutation c.3509G > C (p.Gly1170Ala), two novel *COL4A4* missense mutation c.668G > T(p.Gly223Val), c.826G > A(p.Gly276Arg) and a novel *COL4A5* missense mutation c.2605G > A(p.Gly869Arg). A novel *UMOD* missense mutation c.686 T > A (p.Met229Lys) was also identified.

Type IV collagen alpha chains form a network of α1α1α2, α3α4α5 and α5α5α6 helical heterotrimers that form the mature GBM. Abnormalities in α3α4α5 alters the expression of the other two chains, which affects trimer formation in the GBM [17, 18]. The molecular conformation of the collagen triple helix imposes stringent amino acid sequence constraints and requires a (GLY-X–Y) (n) repeat pattern and a high content of sub amino acids. The most common pathogenic variant is a single base substitution that leads to a glycine residue substitution, which breaks the GLY-X–Y repeat pattern. The single glycine substitution destabilizes the triple helix through a local break in the hydrogen bond and creates a discontinuity in the alignment of the helix [19]. In this study, nine of the 11 pathogenic variants occurred in the GLY-X–Y repeat sequence. The *COL4A3* missense mutation (p.G1170A), the *COL4A4* missense mutations (p.G223V, p.G466R) and the *COL4A5* missense mutations (p.G869R, p.G787R) are all glycine substitutions in the GLY-X–Y repeat sequence. The *COL4A3* nonsense mutation in Family 7 (E405*) occurs at the second position of the GLY-X–Y repeat and the *COL4A4* frameshift mutation in Family 8 (p.P502Lfs*151) occurs at the third position of the repeat. The *COL4A4* nonsense mutation in Family 7 (p.R1377*) causes a truncation that is not within the repeat. However, a large number of repetitive sequences are present downstream of this truncation. Overall, with the exception of the *COL4A4* missense mutation in Family 8 (p.R1682W), all the pathogenic variants were associated with GLY-X–Y repeat sequences, which would cause a change in protein conformation, as verified by our 3D structure predictions.

With the development of genetic technology, more digenic mutations in the *COL4A3* and *COL4A4* genes have been identified and reported [20, 21]. Given the frequency of autosomal dominant Alport syndrome, X-linked form is no longer though to account for 85% of cases of AS. In this study, there were two families carried mutations in two genes. Family 2 had two monogenic missense mutations in the *COL4A3* and *UMOD* genes, whilst Family 3 had a *COL4A3* missense mutation and a heterozygous *COL4A4* frameshift mutation. Digenic mutations are often considered to cause more severe phenotypes than single gene mutations. This phenomenon was also found in the two digenic mutant families in this study. Family 2, II3 has decreased eGFR but with no hematuria or proteinuria, which was consistent with the clinical presentation of UMOD mutation-induced, autosomal dominant tubulointerstitial kidney disease (ADTKD) [22]. Serum uric acid and renal tissue uric acid also supported UMOD-induced ADTKD. In family 2, patient I-2 and II-2 carrying “only” the UMOD variant both died of uremia. II-3 has decreased eGFR but with no hematuria or proteinuria. The proband III-1 has a higher blood uric acid level, sometimes her blood uric acid level is greater than 600umol/l, and she usually takes oral medication to lower uric acid, such as febuxostat, and can maintain normal levels of uric acid. The deposition of crystalline substances in renal tissue is likely due to uric acid.

In AS caused by pathogenic variants in the *COL4A3* and *COL4A4* genes, there is a high variability in patient symptoms. Some patients with a mutation, as identified by genetic testing, are asymptomatic, have isolated hematuria or present with renal failure. This presents a major obstacle for the diagnosis of AS and is highly controversial for the definition of the disease [16, 23–26]. In previous reports of *COL4A3* and *COL4A4* mutations, the terms "familial benign hematuria (BFH)" and "thin GBM disease" were used [27, 28]. In this study, patients with *COL4A3* or *COL4A4* mutations showed thin GBM, by EM, and indicators of loss of renal function in the form of increased urinary protein and decreased eGFR, in addition to hematuria. This demonstrates that the disease is not benign, as previously defined, when it is caused by *COL4A3* and *COL4A4* mutations. In this study, BFH, TBMN and others were uniformly referred to as AS.

It is generally accepted that pathogenic variants contribute to AS and the severity and progression of clinical symptoms correlate with the type of mutation. The differential impact of various mutations may be an important predictor of disease severity and frameshift mutations are often considered to be the disease-causing mutation [29]. Family 3 had a *COL4A4* frameshift mutation (p.P502Lfs*151) that appeared to have a variable phenotype. The two mutations of II-4 were inherited from I-1 and I-2 respectively and located in different chromosomes (in trans), and the mode of inheritance was AR. Two members of the family, I-1 (64-year-old, male) and III-1 (7-year-old, male), carried the frameshift mutation but their renal function was normal and they did not develop progressive hematuria. In contrast, II-2 (38-year-old, female) developed obvious hematuria. Similarly, in Family 6, Family 8 and Family 9, patients with the same pathogenic variant exhibited varying degrees of clinical symptoms. This suggested that there are differences in the severity of clinical symptoms in AS, which may be caused by differences in gender, epigenetic material, environmental factors, smoking and other lifestyle habits [30]. This is consistent with Savige J, Torra R et al. [12, 25, 31, 32], who reported that AS, which included thin GBM nephropathy, has high clinical variability when caused by mutations in the *COL4A3* and *COL4A4* genes. It needs to be emphasized that COL4A4 delC frameshift mutation in I-1 does not produce a clinical phenotype, but in II-2 has full field of erythrocyte, it is a clinically and genetically heterogeneous group of disorder, and is also possible that there is some RNA mediated non sense disease taking place that manages to remove the frameshifted peptide, thereby limiting the translation of abnormal proteins to avoid leading to diseases.

To date, multiple different *COL4A5* mutations have been identified and include deletions, frameshift mutations, nonsense mutations, splice mutations and missense mutations. Mutation of *COL4A5* can interfere with the synthesis of the type IV collagen α5 chain and result in the loss of expression of the type IV collagen α3α4α5 chain in the GBM. The pathogenicity of these mutations is mostly predicted by tools such as SIFT [16]. However, In vitro confirmation of *COL4A5* gene mutations has been rarely reported. In vitro experiments on the *COL4A5* missense mutations (p.G869R, p.G787R) have not been reported. In this study, we found that mRNA levels were significantly lower in the presence of the *COL4A5* c.2359G > C or c.2605G > A mutation in HEK-239 T cells, than in cells without the mutation. Subcellular localization was not altered by either missense mutation and both the wild-type and mutations were expressed in the cytoplasm. This is further evidence that the *COL4A5* missense mutations, c.2359G > C, p.G787R and c.2605G > A, p.G869R, are deleterious. Unfortunately, due to the limitation of antibodies, we did not carry out quantitative analysis of the *COL4A5* protein. The pathogenic mechanism of *COL4A5* mutation needs further study.

In conclusion, this study broadens the scope of *COL4A3, COL4A4, COL4A5* variants even if for most of these novel mutations further clinical data and functional studies are needed to define the pathogenicity. It could have an important implication for the diagnosis of AS and for genetic counselling.

### Supplementary Information

Supplementary Material 1.

## Data Availability

All data generated or analysed during this study are included in this published article. The datasets generated during the current study are available in the BankIt repository, https://www.ncbi.nlm.nih.gov/. The original contributions presented in the study are included in the article, further inquiries can be directed to the corresponding authors.

## Abbreviations

ADAS: Autosomal Dominant Alport Syndrome
ADTKD: Autosomal dominant tubulointerstitial kidney disease
ARAS: Autosomal Recessive Alport Syndrome
AS: Alport Syndrome
EM: Electron Microscopy
GBM: Glomerular Basement Membrane
HE: Hematoxylin-Eosin Staining
KRT: Kidney Replacement Therapy
PAS: Periodic Acid-Schiff Stain
PASM: Periodic Acid-Silver Metheramine
PolyPhen: Polymorphism Phenotyping
WES: Whole Exome Sequencing
XLAS: X-Linked Alport Syndrome
TBMN: Thin Basement Membrane Nephropathy
