# Supplementary figures and images for: Four novel mutations identified in the COL4A3, COL4A4 and COL4A5 genes in 10 families with Alport syndrome
Source: BMC Med Genomics. 2024 Jul 8;17:181. doi: 10.1186/s12920-024-01953-0 (PMC11229269; doi:10.1186/s12920-024-01953-0)

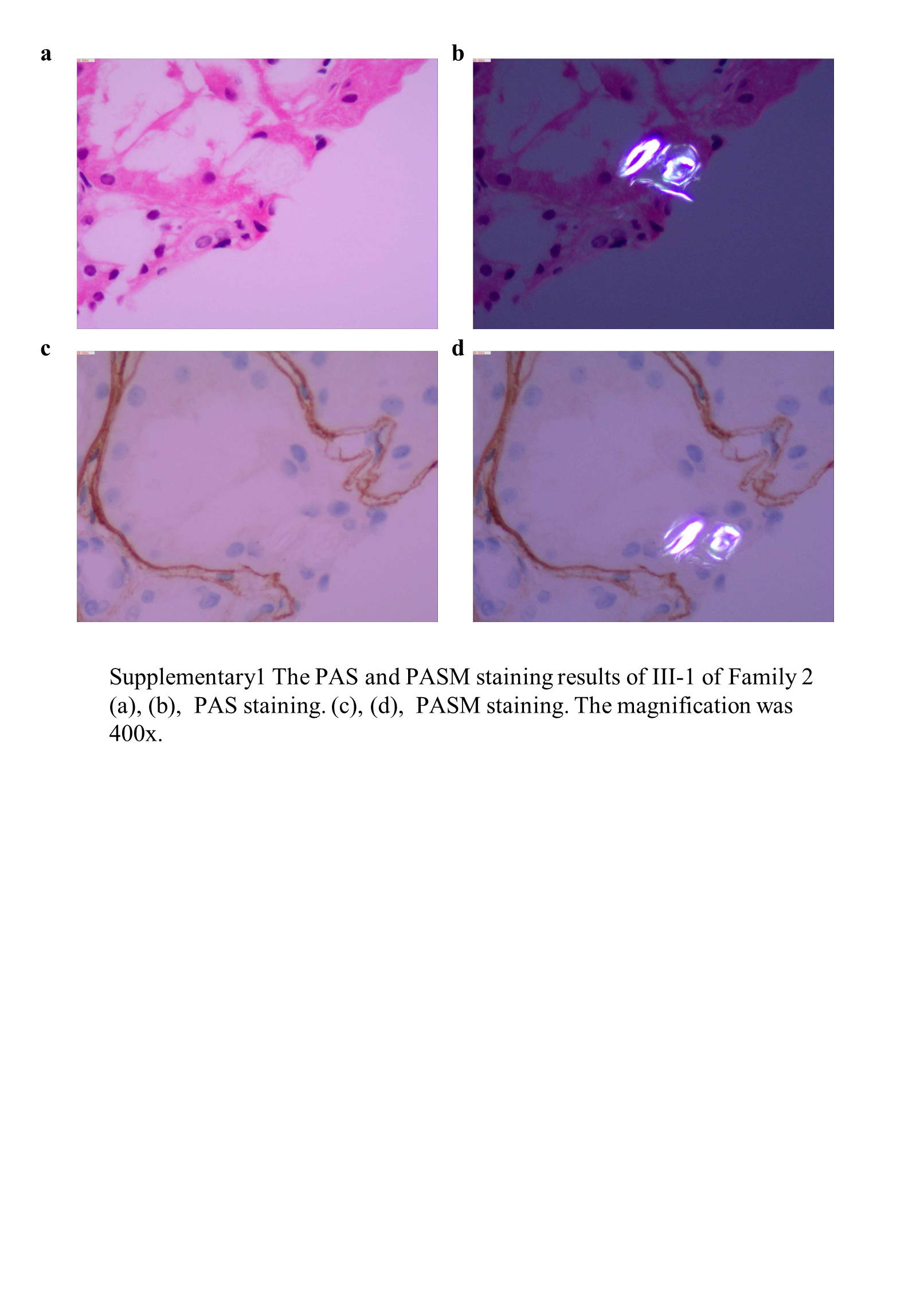

Supplement: Supplementary file 1 — Supplementary Material 1. [file 12920_2024_1953_MOESM1_ESM.tif]
